# Investigational New Drug‐enabling studies in a human vessel‐chip: Are we there yet?

**DOI:** 10.1002/btm2.70129

**Published:** 2026-03-17

**Authors:** Ankit Kumar, Ranganath Maringanti, Tanmay Mathur, Jun‐ichi Abe, Nhat‐Tu Le, Yiwei Xiao, Guangyu Wang, Anahita Mojiri, John P. Cooke, Abhishek Jain

**Affiliations:** ^1^ Department of Biomedical Engineering Texas A&M University College Station Texas USA; ^2^ Department of Cardiology The University of Texas MD Anderson Cancer Center Houston Texas USA; ^3^ Department of Cardiovascular Sciences Houston Methodist Research Institute Houston Texas USA; ^4^ Department of Medical Physiology, College of Medicine Texas A&M Health Science Center Bryan Texas USA

**Keywords:** atherosclerosis, drug testing, personalized medicine, thrombosis, vascular engineering, vascular transcriptomics, vessel‐on‐chip

## Abstract

The US Food and Drug Administration (FDA) Modernization Act 3.0, and the announcement of an National Institutes of Health (NIH)‐wide Office of Research Innovation, Validation, and Application has increased funding for, and encouraged development of, human avatars for disease modeling and drug discovery. This pivotal change has sparked excitement among engineers, scientists, and industry stakeholders to utilize microphysiological systems—also known as organ‐chips—as viable alternative platforms that may be alternatives to animal models in replicating human pathophysiology. The promise of such systems is that they will be more predictive of clinical responses to novel therapeutic interventions. Furthermore, such systems lend themselves to relatively more patient‐specific approaches. These human chips might support precision medicine by predicting response to drugs and therapies—in early clinical trial phases or perhaps even at the bedside. However, for vascular avatars to be useful in preclinical drug development or in clinical trial refinement, several technical, scientific, and educational barriers remain to be addressed. This review highlights the current advancements, potential, and challenges in leveraging vessel‐chip technologies to accelerate vascular medicine and drug discovery, raising the prospect of more rapid FDA investigational new drug approvals and efficient clinical trials.

## Graphical abstract

The human microphysiological systems of blood vessels (vessel‐chips) have shown immense prospective value in reproducing critical aspects of vascular physiology, pathology, and assessment of chemicals and drugs. This review consists of an in‐depth discussion of the state‐of‐the‐art in vessel‐chips, their potential to enable the discovery of precision interventions and translational cardiovascular research.

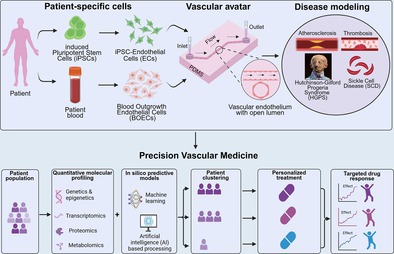

**Abbreviations**
BBB: blood–brain barrier
BFP: blue fluorescent protein
CDI: cellular dynamics incorporated
CT: computed tomography
DAPI: 4′,6‐diamidino‐2‐phenylindole
ERK: extracellular signal‐regulated kinase
GFP‐HUVECs: green fluorescent protein – human umbilical vein endothelial cells
GMP: good manufacturing practice
hAoSMC: human aortic smooth muscle cells
HUVEC: human umbilical vein endothelial cell
ISO: International Organization for Standardization
ISTAND: Innovative Science and Technology Approaches for New Drugs
KDR: kinase insert domain receptor
mRNA: messenger ribonucleic acid
NASA: National Aeronautics and Space Administration
NIH: National Institutes of Health
NINDS: National Institute of Neurological Disorders and Stroke
PDMS: polydimethylsiloxane
PDZ: post‐synaptic density protein 95 + drosophila disc large tumor suppressor + zona occludens 1
PECAM‐1: platelet endothelial cell adhesion molecule‐1
PI3K: phosphoinositide 3‐kinase
SMAD: suppressor of mothers against decapentaplegic
THP‐1: Tohoku Hospital Pediatrics‐1
TNF‐α: tumor necrosis factor alpha
USFDA: United States Food and Drug Administration
VEGF: vascular endothelial growth factor

**Translational Impact Statements**

This review examines human vessel‐chips as microphysiological platforms replicating the cardiovascular environment. These vascular avatars use patient‐specific cells to model atherosclerosis, thrombosis, and sickle cell disease with higher predictive power than animal models. By integrating multi‐omics and artificial intelligence, researchers can analyze how age, sex, and race influence clinical responses. Their relevance has surged under the Food and Drug Administration (FDA) Modernization Act 2.0/3.0. The research into their progress suggests that through the FDA Innovative Science and Technology Approaches for New Drugs (ISTAND) program, vessel‐chips may possibly be validated drug development tools to accelerate approvals.

## 1 INTRODUCTION

Cardiovascular diseases (CVDs) and their associated vascular complications account for the majority of patient deaths globally.^1^ According to a 2015 study by the American Heart Association (AHA), nearly 100 million patients (41.5% population) experienced some form of cardiovascular complication, and unfortunately, this number is projected to rise nearly to 150 million by 2035.^2^, ^3^ The economic burden of treating and managing these complications has also escalated in recent years. Recent estimates suggest that direct and indirect costs of CVDs in the United States reached approximately $555 billion annually. This figure is expected to double, with projections indicating that total treatment costs could reach nearly $1.1 trillion.^4^, ^5^ Moreover, the disparities in CVD prevalence are evident in the United States is also nuanced by its disproportionate impact on different communities, with African Americans bearing a higher disease burden.^6^, ^7^

One key factor contributing to the dramatic rise in the treatment costs of CVDs in recent years is the inconsistency between data obtained from animal studies and results from human clinical trials.^8^, ^9^ Clinical trials of a new cardiovascular therapeutic are costly and time consuming. The definitive endpoints for such trials are often death and/or major adverse cardiovascular events (MACE). Importantly, clinical trials require significant time and numbers of subjects. Thus, there is scope for innovations in preclinical models that offer promise to accurately reflect human disease and test therapeutic interventions with higher predictive power. However, animal models, in many cases, do not reliably mimic the vascular physiology, hemodynamics, and immunological responses observed clinically.^10^, ^11^, ^12^ Importantly, the inability of animal models to recapitulate the heterogeneity observed in human populations further reduces their predictive value.^13^, ^14^ In addition, there are ethical reasons to reduce the use of animals, replacing them with other New Approach Methodologies (NAMs) to strengthen preclinical models that are physiologically relevant and humane. The Food and Drug Administration (FDA) Modernization Act 2.0/3.0 and the announcement of an National Institutes of Health (NIH)‐wide Office of Research, Innovation, Validation, and Application (ORIVA) have accelerated the development of more accurate and predictive preclinical platforms for drug discovery.^8^, ^15^ This has led to a surge in the use of organoids, microphysiological systems (MPS), or organ‐chips that are three‐dimensional (3D) models created from human cells. The MPS platform is designed to reproduce human tissue function that could significantly accelerate preclinical discovery and validation of therapeutics. There is a growing interest to develop fully integrated vascular system components within the MPS community,^16^, ^17^, ^18^, ^19^ as they play a critical role in regulating tissue health and transporting cells, proteins, nutrients, drugs, and waste. Additionally, organ‐chips could selectively dissect the multifactorial determinants that contribute to the onset and progression of vascular diseases such as atherosclerosis. Thus, vessel‐chip platforms are ideal for isolating the specific roles of different cells and their molecular interactions during disease progression. Due to their significant potential, vascular organ‐chips or vessel‐chip models have garnered significant interest recently in research and the pharmaceutical industry^20^, ^21^, ^22^ as surrogate models of human blood vessels.^23^, ^24^, ^25^, ^26^ These humanized vessel platforms offer several technical advantages over existing vascular in vivo and in vitro models. Contemporary vessel‐chip technology has shown promising mimicry of physiological tissue microenvironments enabling real‐time observation of vascular function. In addition to real‐time analysis using immunofluorescence imaging and onboard biochemical sensors, the recent integration with computational and artificial intelligence (AI) based analysis^27^ has made these systems significantly more useful, user independent, and added more predictive power. Further, these platforms engineered as a bottoms‐up approach can independently control fluid dynamics and complex physical and biochemical interactions within complex microenvironments with relatively more ease than animal models.^28^, ^29^ One of the most exciting and transformational aspects of these models is their ability to be cultured with primary patient‐derived vascular cells including endothelial cells (ECs) and vascular smooth muscle cells (VSMCs). Alternatively, one can use induced‐pluripotent stem cell (iPSC) derived vascular cells (e.g., iPSC‐ECs and iPSC‐VSMCs), incorporating diverse population‐based or patient‐specific vascular cues observed clinically.^30^, ^31^, ^32^ Advancements in sequencing techniques, such as global and single‐cell RNA sequencing methods, have significantly improved our understanding of molecular biology from vessel‐chip studies.^33^, ^34^ The ability to investigate the transcriptomic landscape and identify unique pathways using RNA sequencing, when combined with the 3D microenvironmental cues provided by vessel‐chip, can significantly enhance the predictive power of these microphysiological models. The data obtained by converging stem cell biology and omics approaches with vessel‐chip can inform individual patient responses to stressors or drugs, which currently require assessment in clinical research. The more recent entry of AI and machine learning (ML) based analytics has further paved the way for novel innovations and rapid translation of such systems into predicting clinically relevant outcomes. However, even though the promise of vessel‐chip is compelling, these promising possibilities may only be realized after overcoming challenges related to standardization, validation, reproducibility, and end‐user training.

**FIGURE 1:**
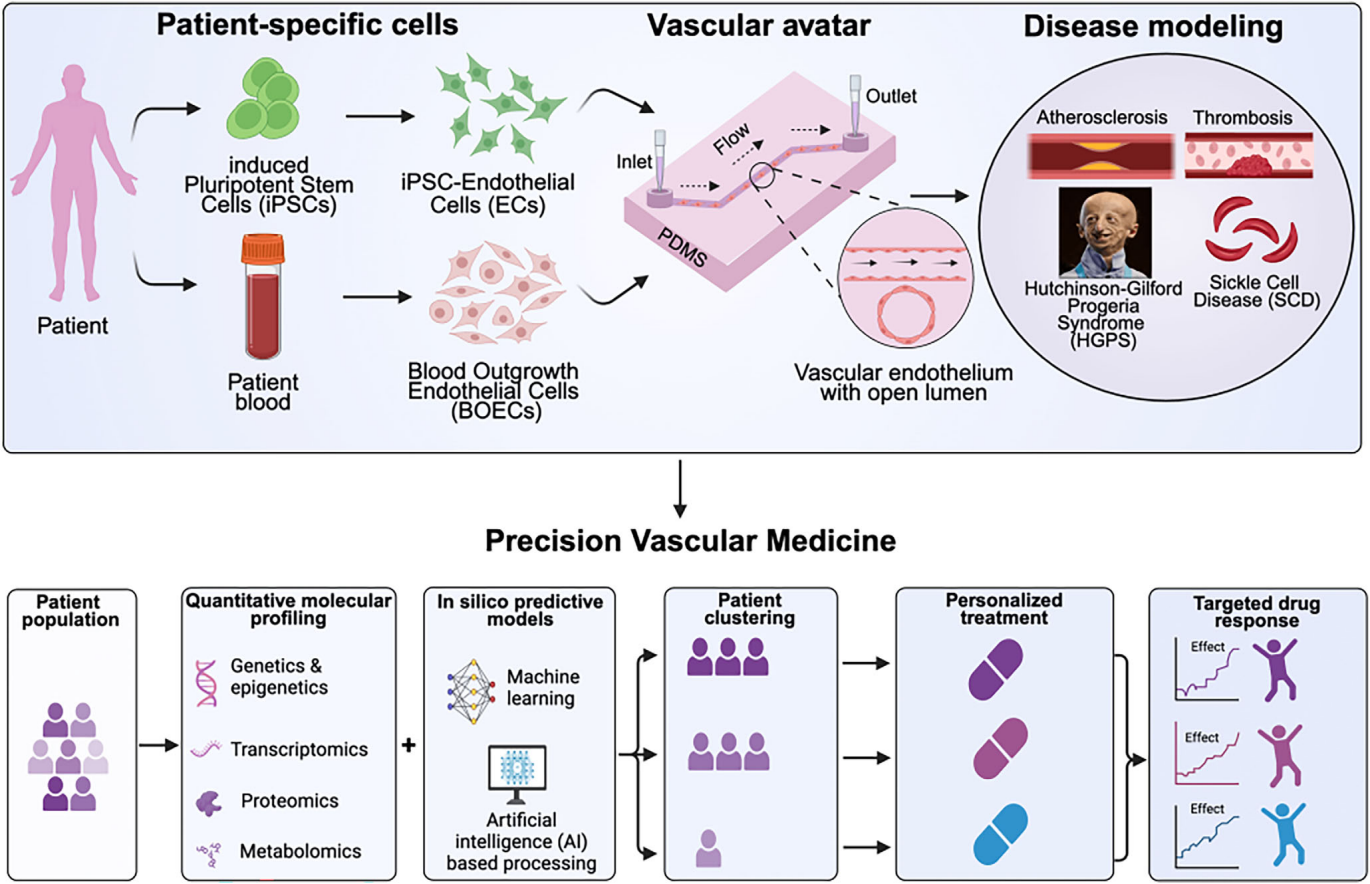
Vascular avatars for precision medicine. The schematic conceptual figure illustrates the development and application of vascular models for disease modeling and therapy using patient‐derived cells and tissue. Patient cells are sourced either as blood‐derived blood outgrowth endothelial cells (BOECs) or reprogrammed into induced‐pluripotent stem cells (iPSCs) and further differentiated into vascular cells (iPSC‐ECs or iPSC‐vascular smooth muscle cells). These cells are cultured within a microfluidic vascular chip system forming a perfusable lumen lined with endothelium—termed a “vascular avatar.” These avatars enable in vitro modeling of vascular diseases such as atherosclerosis, thrombosis, sickle cell disease (SCD) and Hutchinson‐Gilford Progeria Syndrome (HGPS). Integration of patient‐specific avatars with multi‐omics molecular profiling (genomics, transcriptomics, proteomics, metabolomics) and artificial intelligence (AI)‐driven in silico predictive models facilitate patient stratification and clustering. This supports precision vascular medicine by enabling targeted treatment regimens and drug responses.

In this review, we focus on the various applications of vessel‐chip models in studying cardiovascular abnormalities. We discuss: (1) advances in modeling atherosclerosis, thrombosis, and endothelial dysfunction observed in genetic disorders like sickle cell disease (SCD), Hutchinson‐Gilford Progeria syndrome (HGPS); (2) the potential and impact of combining machine‐derived cells with vessel‐chip technology; (3) alternate approaches for incorporating patient‐derived cells such as blood‐derived outgrowth endothelial cells (BOECs); (4) the potential impact of vessel‐chip techniques in modeling age‐, sex‐, or race‐based biological effects observed among the CVD patient cohorts; and (5) the opportunity to integrate omics and ML techniques with vessel‐chip platforms to streamline the drug discovery pipeline (Figure 1). Finally, we address technical barriers and opportunities in vessel‐chip translation and offer our perspective on how to position vessel‐chip platforms as drug development tools for supporting investigational new drug (IND) submissions.

## 2 HUMAN VESSEL‐CHIP MODELS OF VASCULAR DISEASE

### 2.1 Atherosclerosis

Atherosclerosis is a multifocal chronic disease characterized by vascular remodeling and plaque formation^35^ resulting from complex interactions between various vascular and immune cell types.^36^, ^37^ Atherosclerosis could be macrovascular (forming in arteries) and microvascular (forming in arterioles and capillary beds, also known as arteriosclerosis),^38^, ^39^ with distinct pathogenesis processes (Table 1). Accurate representation of the disease and potential therapeutic measures require faithful modeling of these cell types and their relationships, vascular tissue microenvironments including the structure and geometry of the vessels at the site of stenosis, and the relevant forces and factors external to the microenvironment. Endothelial dysfunction and compromised barrier integrity are central hallmarks of atherosclerosis.^40^ The initiation and progression of the disease is also highly dependent on systemic and local immune response involving complex behaviors of monocytes and macrophages. Medial layer smooth muscle cells (SMCs) are another critical component of the vascular wall that undergo significant phenotypical changes during atherosclerosis—resulting in reduced contractility, enhanced proliferation and migration, and transdifferentiation into macrophage‐like cells.^41^, ^42^ Alterations in vascular SMCs, and their interactions with endothelial and immune cells, critically influence the onset of atherogenesis, progression of disease, and stabilization or destabilization of atherosclerotic plaques.^43^, ^44^ Disturbed hemodynamic patterns such as low or oscillatory shear stress are known to impair endothelial function, thereby promoting plaque initiation and progression.^45^ Several clinical risk factors—most notably elevated high levels of low‐density lipoprotein cholesterol (LDL‐C) levels,^46^ hypertension, chronic kidney disease, aging, and hyperglycemia—have been strongly associated with increased susceptibility to atherosclerosis.^47^ Despite these associations, the mechanistic links between individual risk factors and specific pathological features remain poorly defined. Moreover, the complexity of the atherosclerotic microenvironment and its interplay with these risk factors is compounded by substantial variability across patients.

**TABLE 1:** Distinct pathophysiological paradigms of macrovascular and microvascular atherosclerotic disease in humans.

Feature	Macrovascular atherosclerosis	Microvascular atherosclerosis
Location	Arteries (>1 mm diameter)	Arterioles and capillaries (<300 μm)
Primary lesion	Lipid‐laden plaque, eccentric lesion with fibrous cap.	Concentric medial thickening and capillary rarefaction.
Acute event	Plaque rupture leading to thrombotic occlusion and finally myocardial infarction.	Gradual microvascular obstruction leading to ischemia (no acute event).
Driving risk factors	Hyperlipidemia, hypertension, smoking.	Hyperglycemia, oxidative stress, mitochondrial dysfunction.
Inflammatory mechanisms	Monocyte recruitment, foam cell formation, plaque‐resident innate and adaptive immune activation.	Endothelial activation and NO depletion, ROS production, medial smooth muscle cell loss and fibrinoid necrosis.
Flow physiology	Focal stenosis causes proximal high shear and distal low oscillatory shear.	Microvascular obstruction and rarefaction reduces volumetric flow to the tissue.
Clinical manifestation	Acute coronary syndromes, Ischemic stroke, peripheral artery disease.	Microvascular angina, heart failure with preserved ejection fraction (HFpEF), Cerebral small vessel disease.

Abbreviation: NO, nitric oxide, ROS, reactive oxygen species.

**FIGURE 2:**
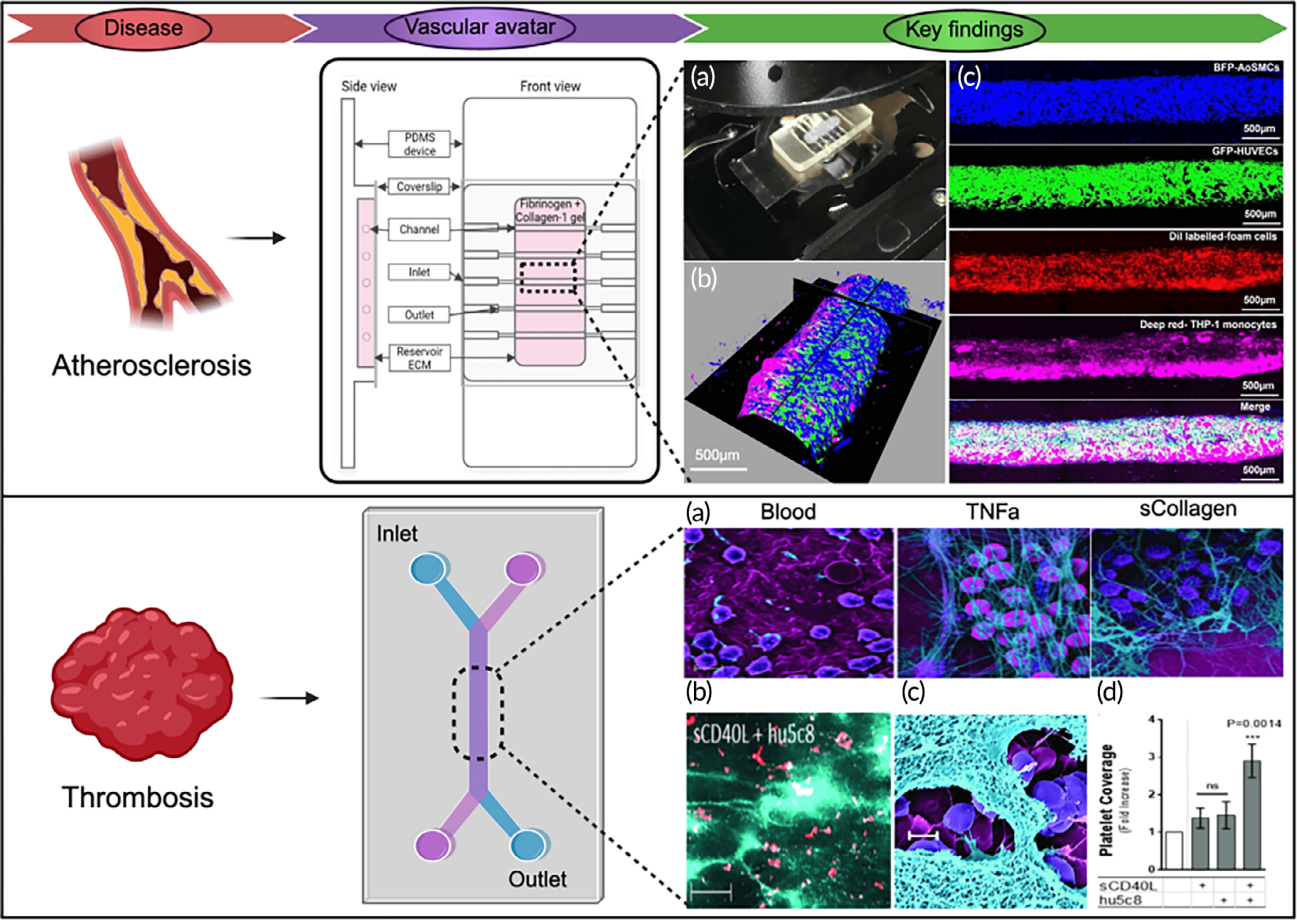
Disease‐specific vascular avatars reveal key mechanisms of atherosclerosis and thrombosis. An illustration of advanced organ‐on‐chip platforms tailored to model human atherosclerosis and thrombosis using microfluidic vascular avatars. Top panel—*Atherosclerosis modeling*: A schematic (center) shows the side and front views of a perfusable PDMS‐based chip embedded with a fibrinogen‐collagen I gel and endothelial‐lined channels mimicking early atherosclerosis.^58^ Panel (a) shows a live chip setup during imaging. Panel (b) depicts a 3D reconstructed image of the atherosclerotic vessel perfused with THP‐1 monocytes.^58^ Panel (c) shows maximum projection image of fluorescent labeling of major vascular components: Smooth muscle cells (Blue Fluorescent Protein (BFP)‐AoSMCs, blue), endothelial cells (Green Fluorescent Protein – Human Umbilical Vein Endothelial Cells (GFP‐HUVECs), green), foam cells (Dil, red), and monocytes (Deep red THP‐1, magenta), with a final merged image highlighting the spatial organization mimicking human atherosclerotic plaque formation. Bar = 500 μm.^58^ Bottom panel—*Thrombosis modeling*: The schematic (center) shows a converging bifurcated chip design consisting of inlet and outlet ports with an endothelialized lumen subjected to flow and prothrombotic stimuli.^22^ Panel (a) shows ultrastructure of thrombus formation under various conditions: Whole blood perfusion, Tumor Necrosis Factor Alpha (TNF‐α) exposure, and soluble collagen (sCollagen). Panel (b) presents platelet aggregation (red) and fibrin (cyan) using sCD40L and the inhibitory antibody Hu5c8. Bar = 100 μm. Panel (c) shows a high‐resolution image of microthrombi rich in fibrin via scanning electron microscopy. Bar = 5 μm. Panel (d) quantifies platelet coverage under different experimental conditions, demonstrating statistically significant inhibition of thrombus formation upon hu5c8 treatment.^22^ These advanced models demonstrate the power of vascular avatars in recapitulating complex disease‐specific features and evaluating targeted anti‐thrombotic therapies. ECM, extracellular matrix.

Recently, advances in vitro organ‐on‐chip (organ‐chip) models have gained traction due to their ability to capture the local macrovascular atherosclerotic microenvironment incorporating key cell types and external stimuli (biomechanical forces, inflammatory molecules, etc.). Recent organ‐chips that are easy to manufacture have successfully recapitulated features of early‐stage atherosclerosis by exposing endothelial monolayers to atheroprone stimuli and candidate therapeutics.^48^ These platforms have been further enhanced by incorporating features of macrovascular atherosclerosis including flow patterns and fibronectin‐coated microchannels,^49^ pathophysiological flow patterns,^50^ and lipid‐laden foam cells.^51^ However, despite incremental advances over the last decade or so, these models still have limitations. For example, these systems still do not include the critical influence of geometry‐dependent mechanical cues on endothelial behavior. A few 3D vascular models have gained relatively greater attention due to their ability for reliable mimicking of vascular disease and drug testing. While several models were developed to study endothelial function, angiogenesis, and inflammation,^52^, ^53^, ^54^ there are relatively fewer in vitro human atherosclerosis microfluidic models that also include relevant hemodynamics. In general, these studies incorporate the co‐culture of vascular cells and immune cells mimicking atherosclerotic events. For instance, a tunable‐3D stenosis model using soft lithography, achieving concave‐convex constriction through air pressure regulation to simulate stenotic plaques was applied to study the interactions between circulating immune cells and ECs under disturbed flow and inflammatory factors.^55^ In another demonstration, a capillary burst valve principle along with extracellular matrix (ECM) patterning to shape vessel geometries was employed, enabling quantitative studies on endothelial‐leukocyte interactions.^56^ A medial blood vessel was described that incorporates porous membranes supporting the separate growth of ECs and SMCs, while still facilitating molecular exchange between these cell types. This design provides a valuable tool for investigating cell and molecular mechanisms of atherosclerosis.^57^ More recently, a device for modeling early‐stage atherosclerosis was developed, which made important advances over previous models including incorporation of labeled vascular cells, both ECs and SMCs, circulating monocytes, and oxidized‐LDL loaded macrophages (foam cells). The model also included modified flow conditions creating early lesion morphology with a triple layered structure forming foam cells in sub‐endothelial space. Furthermore, studies using this device have shown that circulating immune cells undergo extravasation into atherosclerotic vessel recruiting toward a foam cell core^58^ (Figure 2). Another advance has been achieved with the extension of self‐healing perfusable microvasculature‐on‐a‐chip culture for over a month.^59^

These examples suggest that existing human macrovascular atherosclerosis models offer substantial potential to elucidate the multifactorial mechanisms underlying disease progression and to evaluate the efficacy of emerging therapeutic strategies. Despite the advances made by these models, they have not adequately recapitulated the distinct pathophysiology of microvascular atherosclerosis,^60^ which affects resistance arterioles and capillaries and is characterized by concentric intimal‐medial thickening, capillary rarefaction, and chronic microvascular dysfunction rather than the classical focal lipid‐laden eccentric plaque formation. The authors highlight this critical yet unmet need since microvascular atherosclerosis accounts for a disproportionately higher disease burden in several patient populations (e.g. heart failure with preserved ejection fraction [HFpEF], diabetic cardiomyopathy etc.) and still remains relatively understudied. Overall, vessel‐chip atherosclerosis models face inherent limitations such as simplified vascular geometries, restricted cell type usage, and absence of multi‐lineage co‐culture systems which hinder their ability to represent the diverse biological determinants involved in the initiation and progression of vascular diseases. Moreover, most existing studies rely on primary cells derived from pooled donor sources. While using pooled cell sources are advantageous due to cost, quick availability and ease of use, it masks population‐specific differences that risk high donor variability during later analytical validation stages. Therefore, there is potential to improve existing vessel‐on‐a‐chip technologies by integrating uniform and patient‐specific sources (e.g., iPSCs) and enhance scalability and modularity to enable fine‐tuning of tissue‐level properties, immune stimulus, and hemodynamic profiles.

### 2.2 Thrombosis

Thrombosis is a multifactorial pathological process driven by endothelial dysfunction, hypercoagulability, and altered local hemodynamics—collectively referred to as Virchow's triad.^61^, ^62^ Although each element of the triad has been extensively investigated individually, the complex interplay among them remains poorly understood. Therefore, effective thrombosis models must recapitulate the individual contributions of these components while enabling systematic investigation of their synergistic interactions. Contemporary vessel‐chip technologies offer the distinct advantage of enabling both independent and integrative manipulation of the three components of Virchow's Triad: vascular hemodynamics, endothelial function, and whole blood composition, positioning them as promising alternatives to conventional disease modeling platforms.^63^, ^64^, ^65^ Under physiological conditions, endothelium exhibits anticoagulant and anti‐inflammatory properties that maintain vascular homeostasis and facilitate efficient blood flow and oxygen transport.^66^ When activated, however, the endothelium initiates pro‐coagulant pathways leading to thrombus formation.^67^ Numerous stimuli may trigger endothelial activation; however, the mechanisms involved in resulting endothelial dysfunction remain to be fully understood. Accurately replicating pathology‐relevant hemodynamic profiles is essential for modeling thrombotic events with translational relevance.^68^, ^69^ Notably, many stimuli implicated in endothelial activation also modulate blood coagulability, though these effects may occur independently and through distinct mechanistic pathways.

Human vascular organ‐chip models can integrate human (or other) ECs, human (or other) blood, and relevant physical forces—including fluid shear stress; known to be essential for normal vascular function and development of thrombosis. Like atherosclerotic vessel‐chip models, thrombosis‐chips primarily feature a monolayer endothelium for assessing ECs' function under various conditions. Vessel‐chip platforms are also well suited for mimicking human hemodynamics and studying their effects on ECs' behavior. Additionally, such platforms offer the flexibility to customize vascular geometries and replicate associated fluid flow patterns observed in vascular diseases. Finally, the integrated capabilities of vessel‐chip make them attractive platforms for modeling sub‐groups within diseased populations, even down to a patient‐specific level by incorporating vascular geometry, cells, and blood.

Various studies have been developed and deployed to investigate thrombosis and therapeutic interventions. Previous research has demonstrated the formation of perfusable, endothelialized microvascular networks, wherein endothelial activation induces in situ platelet adhesion.^70^ Another study demonstrated localized platelet coagulation on thrombogenic surfaces with incomplete endothelial coverage and validated the anti‐thrombotic function of intact endothelium using a vessel‐chip platform.^71^ The controlled engineering of tissue geometries on microfluidic platforms has enabled the development of stenotic vessel‐chip models, which are used to study the progression of atherosclerosis with superimposed thrombosis. A stenosis‐chip model with plaque‐mimicking geometries was shown to recreate disturbed hemodynamic patterns that promote post‐stenotic platelet aggregation.^72^ This work was further advanced using a 3D‐printed microfluidic mold designed to replicate arterial structures measured via computed tomography angiography.^73^ A method for fabricating endothelialized vessel‐chip geometries representing stenosed vessels such as aneurysms and bifurcations through scalable and widely accessible techniques.^74^ Collectively, these studies underscore the importance of reproducing organ and patient‐specific hemodynamic environments associated with complex and diseased vascular morphologies for accurate modeling and assessing atherothrombotic pathophysiology. Vessel‐on‐chip platforms offer considerable promise for modeling deep vein thrombosis (DVT), which remains challenging to study using traditional models. Initial models investigated the effects of hemodynamics within geometries mimicking venous valve structures, that are recognized as primary sites of DVT initiation.^75^, ^76^ However, these models did not incorporate human endothelium, thereby limiting their physiological relevance. A more refined approach introduced a physiologically relevant vein‐on‐a‐chip model composed of primary human ECs and anatomically accurate venous valve architecture derived from Doppler ultrasound data, which were perfused with whole blood.^29^ This system, while providing a more physiologically relevant platform, still requires further optimization to address functional limitations. The venous valve uses a simplified two‐dimensional (2D) or quasi‐3D geometry with stiff valves that do not fully capture the 3D architecture and mechanical properties of native valve cusps. Integration of pulsatile hemodynamics and a contractile skeletal muscle layer surrounding the lumen would also enhance the chip's in vivo resemblance.^77^ However, this would also increase the model's complexity and should be carefully weighed against the model's intended context of use (COU). Meanwhile, organ‐chip systems have proven effective in modeling thrombosis resulting from comorbid conditions such as SCD, diabetes, cancer, and pulmonary pathologies. For instance, pro‐inflammatory mediators released from tumors^78^ and damaged pulmonary epithelium^79^ can induce endothelial activation and adhesion molecule upregulation resulting in subsequent platelet aggregation and coagulation cascades. For example, one study demonstrated^80^ a simulated model of thrombus formation on a vessel‐chip using whole blood derived from SCD patients. Another advanced engineered model was developed featuring a stabilized endothelium that remained responsive to whole blood perfusion and capable of supporting thrombus formation.^81^ Building on these advances, a dual‐channel model was developed incorporating both vascular endothelial and lung epithelial layers separated by a thin membrane, enabling investigation of thrombosis triggered by epithelial‐derived inflammatory stimuli such as lipopolysaccharide (LPS).^82^ Notably, this platform was later adapted to detect off‐target drug‐induced thrombotic side effects that are often missed or underestimated by conventional animal models, underscoring its value as a preclinical tool for thrombosis diagnostics and drug screening^22^ (Figure 2).

Despite notable progress over the past decade in developing vessel‐chip models for thrombosis, further technological refinement is essential to improve their translational potential. For example, one limitation in thrombosis‐specific models lies in the selection of cellular sources.^83^ Often, widely available primary ECs or immortalized cell lines are chosen for convenience and scalability. However, based on anatomical origin, ECs exhibit tissue‐specific phenotypes and functional responses, which are crucial to accurately capturing both physiological and pathological mechanisms. Therefore, technological improvements are required that allow the incorporation of anatomically and functionally relevant endothelial sub‐types for thrombosis modeling and therapeutic evaluation more precisely. Another key limitation in prior studies is the reliance on steady‐state or non‐physiological hemodynamic conditions in perfusion settings. Blood flow in human vessels is spatially and temporally heterogeneous and varies significantly across types of vasculature (arterial, venous, and capillaries), and such variations play a critical role in vascular homeostasis and pathophysiology. However, vessel‐chip models often use simplified or constant flow regimes due to the technical limitations of conventional microfluidic systems. Hence, advanced flow control systems are needed to enable physiologically and pathologically relevant flow patterns for thrombosis modeling. The small channel diameters and high surface area‐to‐volume ratios are advantageous for efficient molecular exchange and reduced sample consumption. However, the low Reynolds number of flows in these channels limits the ability to mimic the non‐laminar flow regimes, which may become contributors to thrombotic initiation and progression in vivo.^84^ Therefore, there is an opportunity for scientists and physicians to collaborate and address these unmet needs. iPSCs have gained significant attention in recent years as promising cellular tools for patient‐specific modeling. While this is a promising approach, the high cost and technical complexity of iPSC derivation, maintenance, and accurate lineage‐specific differentiation remain barriers limiting their widespread utility for clinical translation. One study demonstrated the potential of BOECs as an alternative to iPSCs for generating a thrombosis‐on‐chip model; however, further advances are needed to achieve sufficient scalability and reliability for wider adoption and translation.^85^

Thrombosis has also emerged as a significant health concern for astronauts during and after spaceflight, with increased reports of venous thromboembolism and altered hemodynamics in microgravity. Traditional models fall short in replicating the complex vascular environment under space conditions. Vessel‐chip technology offers a unique opportunity to study thrombosis in a physiologically relevant, human‐based platform that can be adapted for space research. These microfluidic systems mimic endothelial‐lined vasculature under flow, enabling the assessment of clot formation, endothelial activation, and drug responses in microgravity‐like conditions. Although still an emerging application, recent space missions, including those supported by National Aeronautics and Space Administration (NASA)'s Tissue Chips in Space program^86^ have demonstrated the feasibility of sending organ‐on‐chip platforms including vascular models to the International Space Station (ISS) to study human pathophysiology in orbit.

### 2.3 Endothelial dysfunction in diabetes and sickle cell disease

Type 1 diabetes (T1D) is an autoimmune disorder characterized by the destruction of pancreatic beta cells, resulting in insulin deficiency.^87^ While several organ‐chip approaches have been designed to model the pancreatic islet dysfunction,^88^, ^89^, ^90^ endothelial dysfunction represents another critical and prevalent feature of T1D, contributing to the pathogenesis of diabetic vascular complications.^91^, ^92^, ^93^, ^94^ This dysfunction is marked by reduced nitric oxide (NO) bioavailability, elevated oxidative stress, pro‐inflammatory signaling, and abnormal endothelial cell activation. These abnormalities lead to impaired vasodilation, increased permeability, and prothrombotic conditions, all of which predispose individuals to both microvascular and macrovascular complications.^95^, ^96^, ^97^, ^98^ In one study, ECs derived from T1D patients, when cultured in microfluidic vessel‐chips, exhibited impaired growth kinetics and metabolic activity, increased oxidative stress, and enhanced endothelial activation as evidenced by increased platelet adhesion following whole blood perfusion studies.^85^

**FIGURE 3:**
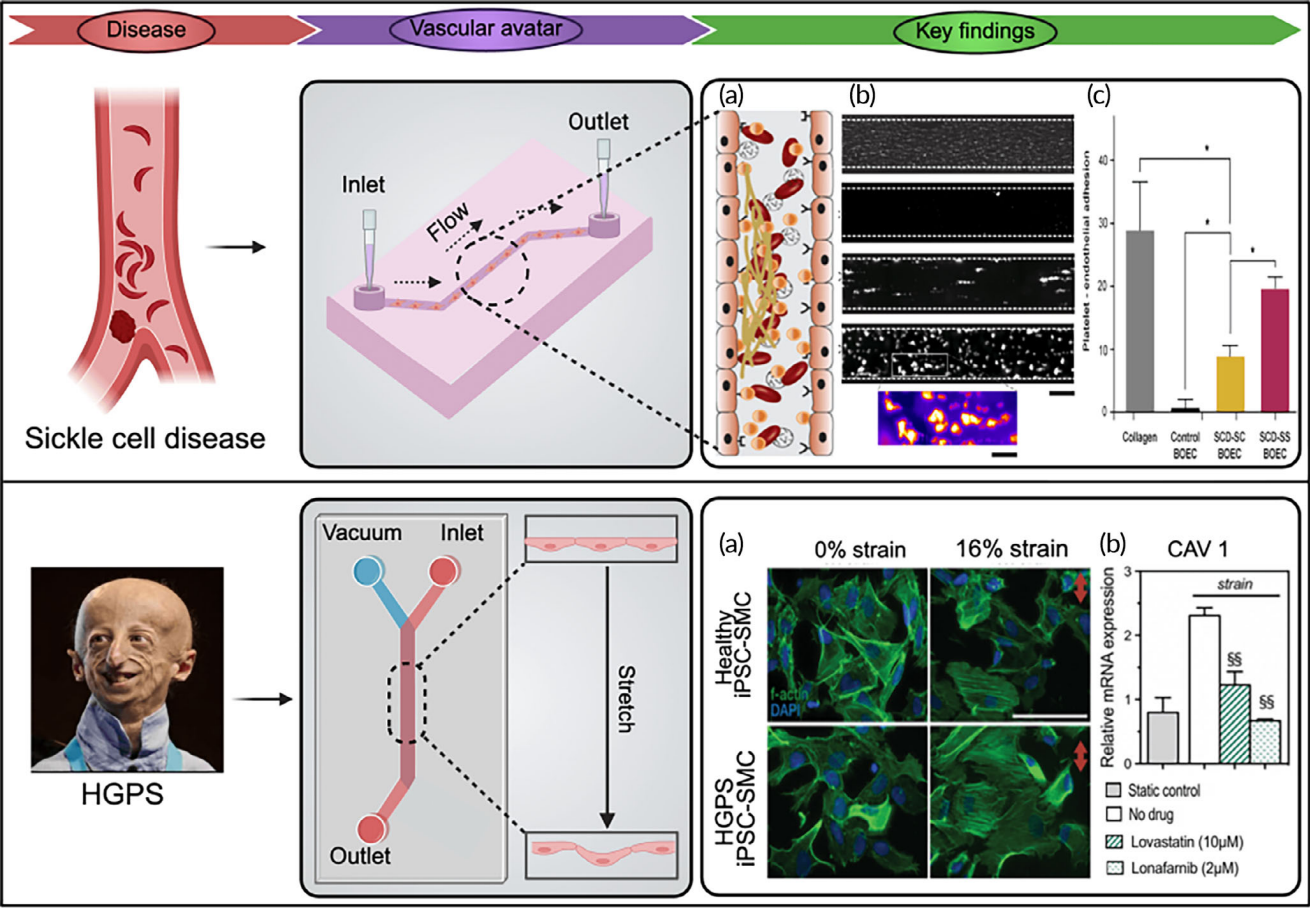
Vascular avatars recapitulate pathophysiology in rare genetic diseases: sickle cell disease (SCD) and Hutchinson‐Gilford Progeria Syndrome (HGPS). This figure presents organ‐on‐chip platforms engineered to mimic vascular dysfunction in two rare genetic disorders—SCD and HGPS—To probe mechanistic insights and therapeutic responses. Top panel—*SCD vascular avatar*: The schematic (center) shows a perfusable microfluidic chip lined with endothelial cells (ECs) derived from blood outgrowth endothelial cells (BOECs), with controlled flow to mimic the in vivo vasculature of SCD patients. Panel (a) shows a cross‐sectional representation of SCD blood flow over the endothelium, illustrating platelet‐endothelial interactions and vaso‐occlusion mediated by sickled red blood cells. Panel (b) provides experimental images capturing platelet adhesion under different conditions control BOEC and SCD BOEC (SCD‐SC and SCD‐SS) vessel‐chip. Bar = 200 μm. Panel (c) quantifies platelet adhesion across various endothelial phenotypes: Control BOECs, BOECs from sickle cell trait (SCD‐SC), and sickle cell disease (SCD‐SS) patients, demonstrating significantly higher adhesion in SCD‐SS conditions, highlighting disease‐specific vascular dysfunction.^106^ Bottom panel—*HGPS vascular avatar*: The schematic (center) illustrates a microfluidic chip design enabling mechanical strain via vacuum actuation, mimicking vascular stiffness and cyclic strain observed in human arteries. Panel (a) presents immunofluorescent images of healthy and HGPS‐derived induced‐pluripotent stem cells (iPSC)‐smooth muscle cells (SMCs) under 0% and 16% strain, showing cytoskeletal organization (F‐actin, green) and nuclear integrity (4',6‐Diamidino‐2‐Phenylindole (DAPI), blue). HGPS cells show abnormal morphology and reduced mechanosensitivity. Panel (b) displays relative Messenger Ribonucleic Acid (mRNA) expression of caveolin‐1 (CAV1), a key mechanotransduction gene, under different strain and drug conditions. Lovastatin and lonafarnib partially rescue CAV1 expression, indicating therapeutic modulation of vascular phenotype in HGPS.^107^ These rare‐disease‐specific vascular avatars reveal distinct endothelial and smooth muscle dysfunction and offer a personalized preclinical tool for therapeutic screening. *p < 0.05, §§*p* < 0.001.

SCD is an inherited hemoglobinopathy marked by the presence of abnormal hemoglobin (HbS), which induces pathological deformation of red blood cells (RBCs) into rigid, crescent or “sickle” shape. Several vessel‐chip models have been developed to investigate microvascular occlusion events initiated by RBC sickling. Some systems quantified the deformability of sickle RBCs,^99^, ^100^ while others assessed the influence of extracellular fluid tonicity on sickling behavior.^101^ A relatively advanced model incorporated a branched microvascular chip offering real‐time monitoring of vaso‐occlusion events using blood samples from sickle cell patients.^80^ Beyond RBC deformability, SCD is marked by chronic hemolysis, releasing free heme that contributes to endothelial injury and coagulation.^102^ Vessel‐chip studies have demonstrated the deleterious effects of elevated heme on endothelial function and RBC behavior. Although endothelial dysfunction is a well‐recognized contributor to SCD pathology, it has been less frequently modeled.^59^ In addition to the RBC sickling and hypercoagulability, endothelial dysfunction is a clinically recognized hallmark—but underexplored feature of SCD pathophysiology.^103^, ^104^ More recent vessel‐chip platforms utilized BOECs from SCD patients, predicting endothelial dysfunction correlating with clinical severity (Figure 3).^105^ These models captured functional heterogeneity among patients, with transcriptomic analysis revealing distinct gene expression profiles associated with endothelial activation. The whole blood perfusion assays further confirmed that ECs from patients with more severe clinical symptoms supported significantly higher levels of platelet adhesion, reinforcing the role of endothelial variability in disease severity. (Figure 3).^105^

### 2.4 Evaluation of endothelial dysfunction in genetic disorders

The use of stem cell‐derived cells has enabled the investigation of rare genetic disorders in the context of vessel‐chip technology. One such disorder, HGPS, is caused by a single mutation in the LMNA gene, leading to the permanent farnesylation of LaminA and the production of an abnormal form called progerin.^108^, ^109^ Progerin accumulation at nuclear envelops causes nuclear lobulation, distortion, and aberrant gene expression. While EC dysfunction is widely recognized in HGPS pathophysiology, recent clinical and mechanistic studies have also revealed that medial SMC loss and dysfunction might represent the primary vascular pathological feature, with downstream effects on EC function.^110^, ^111^ Specifically, progerin accumulation in SMCs leads to excessive apoptosis, reduced contractility, impaired mechanotransduction, and deposition of collagen and ECM in the medial layer, which promote EC dysfunction and endothelial to mesenchymal transition (EndoMT).^112^, ^113^, ^114^

The EC dysfunction is marked by the reduction of NO production and availability, resulting in vasoconstriction, increased oxidative stress, inflammation, and enhanced ECs activation.^115^, ^116^ The crosstalk between SMCs and ECs results in inflammatory responses and the development of atherosclerosis in HGPS patients. To recapitulate these pathologies in vitro, arteriole‐scale vessel‐on‐chip models were constructed using ECs^117^ and SMCs^118^ derived from both healthy individuals and HGPS patients using iPSCs. These engineered vessels effectively recapitulate some of the hallmarks of endothelial dysfunction observed in HGPS, including increased inflammatory cytokines and reduced vasoactivity.

Mechanical cues, such as substrate stiffness and fluid shear stress, are also critical modulators of vascular pathophysiology. Using a mechanotransduction‐on‐a‐chip platform, the behavior of endothelial mechanosensors yes‐associated protein (YAP) and TAZ (transcriptional coactivator with a Post‐synaptic density protein 95 + Drosophila disc large tumor suppressor + zona occludens 1 (PDZ)‐binding motif) was investigated.^119^ Their model recapitulated the nuclear localization of YAP under low shear conditions across substrates with varied matrix stiffness values. In contrast, under high shear stress and increased matrix stiffness, the ECs showed a significant increase in YAP nuclear localization.^119^

Further studies examined the effects of cyclic mechanical strain on iPSC‐derived SMCs from HGPS patients.^107^ (Figure 3). When cultured on a thin PDMS membrane and subsequently subjected to continuous cyclic strain, iPSC‐derived SMCs from Progeria patients exhibited increased DNA damage and a higher presence of inflammatory and senescence markers. Using this model, the authors also demonstrated the efficacy of the first drug candidate designed to treat Progeria, lonafarnib^120^ (Figure 3).

While some existing models include EC‐SMC co‐culture with intimal and medial layers, recapitulation of the characteristic SMC loss and careful investigation of the cellular crosstalk driving cell dysfunction has been lacking. Therefore, future work may benefit from the inclusion of SMC apoptosis and senescence as readouts, measure SMC contraction under physiological cyclic shear conditions, and evaluate multi‐drug therapy regimes that may synergistically work on SMCs and ECs to restore vascular homeostasis.

## 3 THE PROS AND CONS OF COMBINING STEM CELLS WITH VESSEL‐CHIP MODELS

With advancements in in vitro research tools including organ‐chips, there is increasing interest in identifying reliable cell sources capable of meeting the requirements for accurate disease modeling and drug testing.^121^ Primary cells have historically served as the most common source for human‐based in vitro models; however, their use is limited by several key drawbacks. These include limited availability, patient‐to‐patient variability, and challenges in isolating pure populations of specific vascular cell types from heterogeneous samples.^23^

Stem cells represent an important resource for overcoming many of these challenges. iPSCs^122^ provide a renewable and genetically stable source of human cells for human disease models with greater reproducibility compared to primary cells.^123^ Protocols for differentiation of iPSCs into various specific vascular cell types (e.g., tissue‐specific arterial,^124^, ^125^, ^126^, ^127^ venous,^125^, ^128^ and lymphatic^129^ ECs as well as supporting SMCs^130^, ^131^, ^132^ and pericytes^133^, ^134^, ^135^) are continually being refined and expanded. This enables high‐throughput screening, modeling rare or genetically distinct patient conditions, and inclusion of populations typically underrepresented by clinical trials. The implementation of advanced gene editing tools further enhances researchers' abilities to study the effects of genetic mutation on disease and drug response.

The use of patient‐specific iPSCs in organ‐chip systems enables personalized disease and drug testing models for both preclinical development and clinical trial strategies. Organ‐on‐a‐chip platforms using iPSC‐derived vascular cells recreate human‐relevant physiological environments, enhancing the predictive accuracy of drug testing. Importantly, these models can be tailored to reflect diverse demographics and coexisting morbidities. Furthermore, these patient‐mimetic platforms provide translational insights that can guide the recruitment of at‐risk or underrepresented groups in clinical trials, aligning with the FDA's priorities for inclusivity.^136^ Finally, iPSC‐based vascular organ‐on‐a‐chip models can serve as platforms for surrogate endpoint testing during clinical trials. Their accessibility for real‐time monitoring of cell and tissue function provides unique opportunities to confirm disease‐specific responses and mechanistic alterations in correlation with clinical outcomes.

Despite these promising features, some technical and logistical challenges limit the immediate adoption of iPSC‐based vessel‐chip models in drug discovery at a large scale. While effective differentiation is often complex, several strategies could help researchers mitigate these barriers. Pre‐established iPSC repositories such as the Coriell Institute for Medical Research, National Institute of Neurological Disorders and Stroke (NINDS) Human Cell and Data Repository, and Cedars‐Sinai maintain a large collection of iPSC lines spanning healthy controls and disease‐specific populations. Disease‐specific foundations such as the Progeria Research Foundation maintain curated iPSC banks specifically derived from affected patients, eliminating the need for patient recruitment and in‐house reprogramming. For clinical applications, Good Manufacturing Practice (GMP)‐manufactured iPSCs are now available from vendors such as Lonza and Fujifilm Cellular Dynamics Incorporated (CDI). These resources increase accessibility to researchers that lack specialized stem cell biology expertise or institutional infrastructure. However, other limitations remain, such as the reliance of iPSC differentiation protocols on 2D culture, which poorly recapitulates the native 3D structures observed in vivo. Likewise, most differentiation approaches focus on biochemical cues while neglecting biomechanical stimuli, which are crucial for physiologically relevant vascular phenotypes.^137^, ^138^ Additionally, the lack of standardized protocols across laboratories results in batch‐to‐batch variability, ultimately impairing reproducibility. In many cases, different laboratories employ distinct reagents and procedures to culture the same target cell type. Furthermore, protocols for generating new tissue‐specific vascular cell types are underdeveloped. For instance, methods for deriving many venous and lymphatic endothelial cell types remain elusive. Even when protocols claim to achieve differentiation into desired cell types, validation of iPSC‐derived cells remains a significant challenge. The expression of specific markers is an important aspect of confirming cell identity; however, reliance on these markers alone is often insufficient. This necessitates comprehensive multiplexed strategies to confirm the acquisition of target phenotypes.

Collectively, the development of organ‐on‐a‐chip platforms incorporating stem cell‐derived vascular tissues represents a powerful strategy for developing physiologically relevant human disease models. These models support a wide range of applications ranging from mechanistic studies to clinical trial optimization. Advancing standardization of differentiation protocols and establishing robust validation strategies will be essential in building scientific rigor and regulatory acceptance.

## 4 BLOOD OUTGROWTH ENDOTHELIAL CELLS AS DISEASE AND PATIENT‐SPECIFIC VASCULAR CELL MODELS

Tissue biopsies have traditionally been considered the gold standard for isolating primary ECs in literature.^139^ Although minimally invasive biopsies have advanced, tissue biopsies still carry a relatively high risk of vessel and tissue injury due to the need for localized surgical intervention.^139^ Embryonic stem cells (ESCs) or iPSC‐derived cells represent a promising alternative source for obtaining autologous ECs.^140^ However, these approaches often vary significantly in terms of endothelial cell yield and purity, with certain approaches yielding only 6%–16% efficiency.^141^ Furthermore, the exact differentiation pathways and growth factors used in obtaining differentiated ECs differ considerably between research groups.^142^ The technical complexity and need for specialized training to handle stem cell‐derived tissues, coupled with limited feasibility in low‐resource settings, present further challenges to the widespread adoption of these approaches.

Circulating endothelial progenitors (EPCs) present in blood can serve as an alternative strategy for sourcing autologous ECs.^143^ BOECs, a late‐stage subtype of EPCs, can be reliably derived from patient blood through standard density gradient centrifugation techniques.^144^ Studies from our group and others have demonstrated that autologous BOECs express CD31 or Platelet Endothelial Cell Adhesion Molecule‐1 (PECAM‐1), CD34, Vascular Endothelial Growth Factor (VEGFr2) Kinase Insert Domain Receptor (KDR), Von Willebrand factor (VWF), and endothelial nitric oxide synthase (eNOS).^34^, ^85^, ^105^, ^145^ Functionally, they exhibit key phenotypic responses including exogenous shear, stimulation, growth kinetics, and vasculogenic potential comparable to iPSC‐derived ECs.^33^ Our studies with BOECs from patients with SCD and T1D have shown that these cells can effectively mimic the disease‐specific phenotypes in vitro and sensitively distinguish between heterogenous patient populations.^33^, ^34^, ^85^, ^105^ Their ease of isolation and expansion makes BOECs a compelling autologous endothelial cell model for vascular research. Clinicians or pharmaceutical developers can then potentially use BOECs on vessel‐chip platforms to stratify patients based on disease severity, thereby streamlining drug discovery/testing pipelines and moving closer to personalized care.

## 5 EXPLORING AGE, SEX, AND RACE AS BIOLOGICAL VARIABLES IN DISEASE WITH VESSEL‐CHIP

Although the use of patient‐derived cells in in vitro human disease models has received increasing attention, there remains a clear and pressing need to better represent patient diversity in these systems. In fact, the US FDA has required until recently that researchers and pharmaceutical developers submit formal plans to ensure demographic diversity in late‐stage clinical trials. Given the ongoing challenges of clinical trial design, recruitment, and representation, it is essential that patient diversity be addressed throughout the entire translational research and drug development pipeline, including basic science research. While this need exists across all disease areas, disparities in the prevalence and outcomes of vascular diseases among demographic sub‐groups are especially pronounced. Age represents one of the most well‐established risk factors influencing CVD incidence. Broadly, the CVD prevalence increases markedly with age rising from ~40% among individuals aged 40–59 to ~75% in those aged 60–79, and ~85% in populations aged above 80^146^; this trend is largely reflected across major disease subsets.^147^ Sex‐based differences also play a significant role in CVD. Men under 55 experience coronary artery disease at more than twice that of women in the same age group, although this gap narrows substantially with advancing age.^148^ In recent years, increasing attention has been given to disparities in CVD across racial and ethnic groups. Black adults in the United States have a 23% greater mortality rate resulting from heart disease than White adults, and more than twice that of non‐Hispanic Asian populations. Despite these glaring disparities, the underlying mechanisms remain poorly understood—largely due to the inability of conventional disease models to incorporate biological variables such as age, sex, and race across diverse patient populations. Vessel‐chip technologies offer a powerful platform for addressing these gaps by enabling incorporation of human vascular cells derived from demographically diverse patient groups. These systems hold potential for mechanistic studies of disease progression, preclinical drug evaluation, and clinical trial support through patient pre‐screening and surrogate endpoint testing in populations that have historically been underrepresented in biomedical research.

## 6 THE OPPORTUNITY OF COMBINING “OMICS” AND AI WITH VESSEL‐CHIP

**FIGURE 4:**
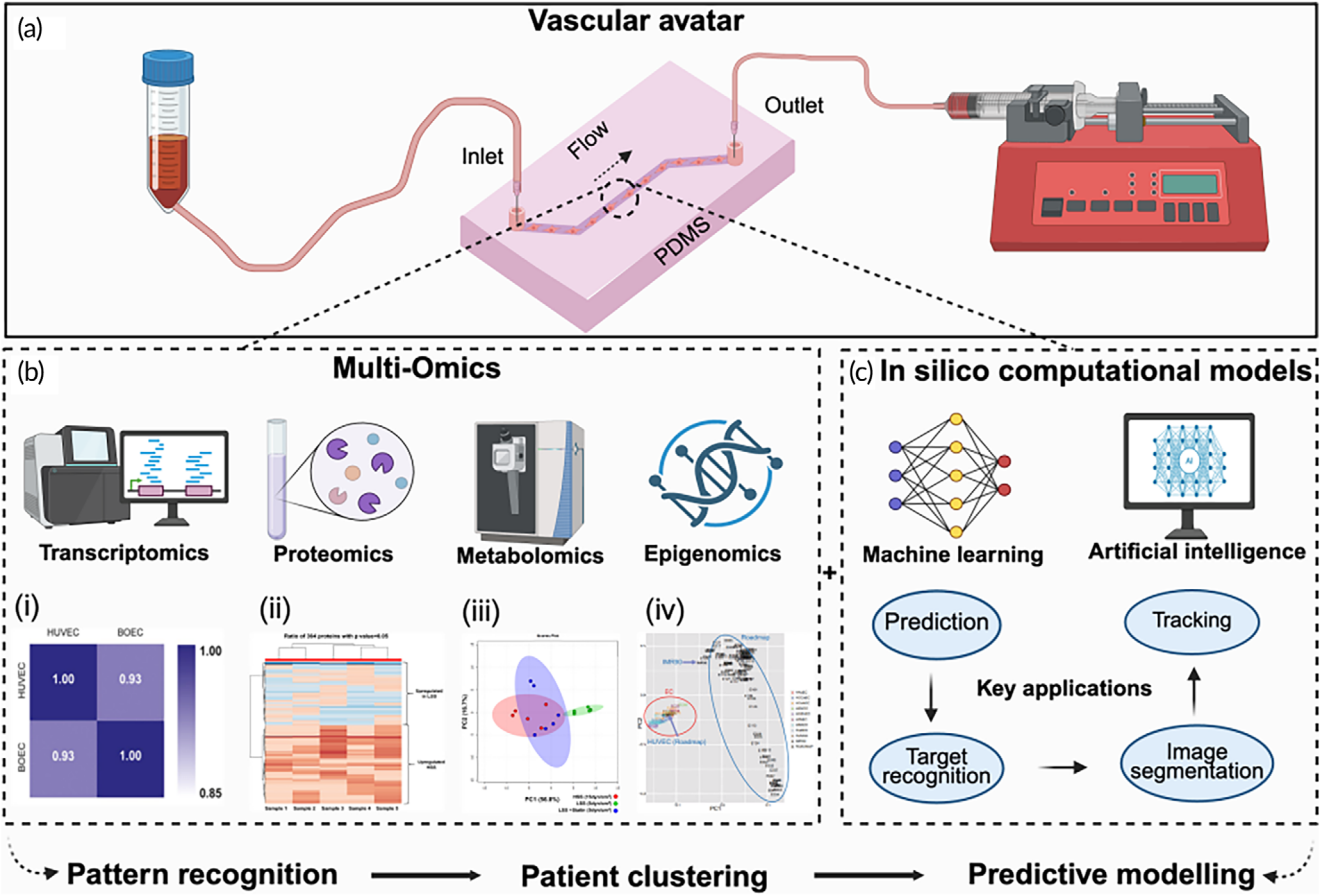
Integration of vascular avatars with multi‐omics and in silico computational modeling for precision medicine. (a) A schematic representation of a vascular avatar system, consisting of a PDMS‐based microfluidic chip perfused with patient‐derived samples to mimic vascular flow. (b) Multi‐omics approaches transcriptomics, proteomics, metabolomics, and epigenomics—are applied to analyze molecular profiles derived from the vascular avatar. Representative data visualizations include: (i) gene expression correlation heatmaps,^34^ (ii) proteomic heatmaps,^155^ (iii) principal component analysis (PCA) of metabolomic data,^155^ and (iv) PCA plot of ECs using H3K27ac from the Roadmap Epigenomics.^156^ (c) In silico computational models, including machine learning and artificial intelligence, leverage the multi‐omics data to perform predictive modeling, target recognition, image segmentation, and tracking. Combining quantitative molecular profiling and in silico predictive tools contributes to pattern recognition and patient clustering supporting the development of personalized therapeutic strategies.

Multi‐omics or “omics” techniques have become increasingly central to vascular OoC research, offering an added dimension of physiologically relevant data to advance in vitro disease modeling tools.^30^, ^149^ These techniques enable analysis of the cellular and subcellular molecular landscapes in both health and disease and allow one to evaluate patient‐specific variations in molecular signaling.^32^, ^150^ Among the most widely applied omics techniques in OoC studies is transcriptomics, which captures gene expression and signaling at the transcriptome level to assess gene expression profiles, uncover previously uncharacterized splicing events, and investigate regulatory mechanisms such as RNA editing, post‐transcriptional processing, and non‐coding RNA activity. Whole‐genome RNA sequencing has been integrated into OoC disease modeling pipelines to assess gene expression profiles, uncover previously uncharacterized splicing events, and investigate gene regulatory mechanisms such as RNA editing, post‐transcriptional processing, and non‐coding RNA activity.^151^, ^152^, ^153^, ^154^ Additionally, our group's transcriptomic profiling of patient‐specific vessel‐chip models has shown the ability to investigate the individual transcriptomic signatures and detect differential expression in key signaling pathways^33^, ^34^, ^105^ (Figure 4).

Proteomics is another critical omics approach relevant to vessel‐chip systems, allowing visualization of the entire protein spectrum in conjunction with vascular pathologies.^157^, ^158^ Techniques like mass spectrometry (MS) or protein‐based arrays can be easily multiplexed with the vessel‐chip pipelines to serve as excellent tools for visualization of the protein‐level changes within the vascular niche. For example, in atherosclerosis, the endothelial proteome undergoes dynamic alterations as ECs transition toward a more atheroprone phenotype^159^, ^160^ (Figure 4). The enhanced physiological fidelity of contemporary vessel‐chip platforms, particularly in recreating tissue architecture and hemodynamics, offers a powerful framework for investigating disruptions in protein signaling mechanisms involved in disease progression.

Additional omics tools, such as metabolomics^155^ and epigenomics^156^ can also be applied through vessel‐chip as surrogates for patient vasculature blood vessels, further amplifying the predictive power of these contemporary models (Figure 4). For instance, in type 2 diabetes where endotheliopathy is a significant hallmark, precise evaluation of the intrinsic metabolic state becomes essential.^91^, ^93^, ^161^ Investigating mechanisms of genetic modifications such as DNA methylation and histone modifications can deepen our understanding of the epigenomic basis of disease progression in conditions like atherosclerosis and diabetes,^50^, ^54^, ^94^, ^158^, ^162^, ^163^ which can be systematically investigated using vessel‐chip systems.

Modern vessel‐chip technologies offer several technical advantages over traditional cell culture models. Their compatibility with real‐time imaging and integration with in‐line biosensors enables high‐throughput generation of large‐scale imaging and biochemical data sets.^164^ With the emergence of ML and AI, these data sets can be leveraged to develop predictive pipelines for data collection, analysis, and interpretation. Several supervised and unsupervised deep learning algorithms have been applied to characterize and measure cellular and tissue‐level physiologies. For example, one study developed a tumor‐on‐chip model with interconnected tumor and immune cell chambers to generate large imaging data sets.^165^ These data sets were analyzed using an unsupervised learning tool, CellHunter,^166^ which quantified parameters such as cell count, average displacement, and dendritic cell mobility. Moreover, integration of imaging data with transcriptomic “big data” (e.g., from RNA sequencing) facilitates the development of classification models that, once trained, can use standard imaging inputs to predict the severity of endothelial dysfunction or vascular inflammation^164^, ^167^ (Figure 4).

## 7 TECHNICAL BARRIERS AND OPPORTUNITIES IN VESSEL‐CHIP TRANSLATION

Several practical challenges hinder the advancement of vessel‐chip platforms as drug development tools.^168^ Their performance for IND screening is closely linked to the materials used to build these systems, and those material constraints, in turn, limit how far the platforms can be pushed toward quantitative pharmacology and IND‐enabling studies. PDMS has been an extremely useful material for academic researchers and even organ‐chip companies because it is easy to prototype, optically clear, gas‐permeable, and biocompatible. Importantly, PDMS vessel‐chip devices have shown promising results, and Emulate, a company that is commercializing PDMS‐made organ‐chips, has now entered the FDA qualification program ISTAND for validation. However, its polymer network has substantial free volume and is hydrophobic, which leads to the sorption of small lipophilic compounds and nonspecific adsorption of proteins to channel walls. Across different studies, PDMS has been reported to deplete certain highly lipophilic molecules from perfusate under continuous flow,^169^ thereby limiting the outcomes of pharmacokinetic studies.^170^

Thermoplastics, such as polymethyl methacrylate, polycarbonate, and cyclic olefin copolymer, offer an alternative to PDMS. Not only do they tend to show lower sorption of hydrophobic molecules than PDMS, but they are also compatible with injection molding and hot embossing and can be manufactured with the high reproducibility expected by industry and regulators.^171^ In addition, microfluidic‐focused polymers such as Flexdym have been developed that retain PDMS‐like elasticity and optical properties while still reducing small‐molecule absorption and meeting International Organization for Standardization (ISO) 10993 and USP Class VI biocompatibility standards. This provides academic researchers that currently rely on soft lithography a more incremental path toward drug‐compatible chips.^172^ A limitation of using thermoplastics, however, is that they are not biocompatible over long durations due to oxygen depletion and tissue hypoxia.

For laboratories with significant infrastructure centered on PDMS devices, surface modification can partially mitigate nonspecific binding, thereby avoiding the need for complete system redesign. Chemical vapor deposition of conformal parylene‐C coating forms a thin, pinhole‐free diffusion barrier within microchannels. Such coatings have been shown to reduce the sorption of small molecules up to six‐fold without compromising optical transparency or biocompatibility.^173^ Hydrophilic polymer coatings, particularly those based on polyethylene glycol such as PLL‐g‐PEG or related chemistries, introduce steric and entropic repulsion at the liquid–solid interface. This approach reduces protein adsorption up to three‐fold.^174^, ^175^ However, the long‐term stability of such coatings for cultures lasting days to weeks remains to be systematically investigated.

Flow control poses an additional technical challenge, as ECs are highly sensitive to both the magnitude and transient characteristics of flow‐induced shear stress.^176^ Conventional peristaltic pumps are widely used due to their accessibility and ease of use; however, they often produce irregular flow outputs, including transient fluctuations, pressure spikes, and significant deviations from the nominal flow rate during extended experiments.^177^, ^178^ Such unstable flow conditions may promote endothelial detachment and induce transitions toward an atheroprone endothelial cell phenotype, complicating the interpretation of drug responses or mechanistic findings.^179^ Pressure‐based controllers, which regulate flow by pressurizing upstream reservoirs, generally provide more stable performance, with reported flow variation reduced to a few percent over prolonged culture. These controllers also enable the programming of steady, pulsatile, or oscillatory shear profiles that more accurately replicate conditions in arteries, veins, or microvessels.^180^ Furthermore, these systems can be integrated with multiplexed fluidic manifolds to perfuse multiple chips at matched wall shear stress and circumferential strain, thereby increasing throughput and ensuring that replicate devices experience comparable mechanical conditions. When combined with cyclic stretch platforms that apply physiologically relevant strain to endothelial–smooth muscle co‐cultures, emerging evidence suggests that the combination of shear and stretch supports tighter junctions, more in vivo–like transcriptional profiles, and enhanced barrier and transport function in several organ‐specific vascular models compared to shear alone,^181^, ^182^, ^183^ although the optimal conditions likely vary by tissue and application.

A third, more biologically oriented challenge is the continued use of generic endothelial sources, such as umbilical vein or aortic ECs, in many vessel‐chip studies, despite well‐documented endothelial heterogeneity across organs and vessel segments.^184^ Variations in endothelial morphology, including fenestrated capillaries in glomerular and endocrine tissues, tight continuous capillaries in the brain and retina, and discontinuous sinusoidal endothelium in organs such as the liver and marrow, are closely associated with differences in vascular permeability and function. These morphological differences are also associated with distinct disease phenotypes. For example, in diabetic nephropathy, filtration is impaired by the loss of glomerular endothelial cell fenestrations and by altered VEGF signaling. In contrast, pericyte dropout and the formation of acellular ghost vessels are observed in diabetic retinopathy, and such lesions are not readily reproduced in Human Umbilical Vein Endothelial Cell (HUVEC) monolayers within simple microchannels.^185^, ^186^ Brain microvascular ECs establish the blood–brain barrier by forming high‐resistance tight junctions, expressing specialized transporters and efflux pumps, and suppressing transcytosis. Replicating this phenotype in vitro generally requires both appropriate endothelial programming and co‐culture with astrocytes and pericytes within a structured microenvironment.^187^

A growing array of tools is now available for constructing more organ‐specific and demographically representative vascular models. Multiple research groups have developed methods to differentiate human induced‐pluripotent stem cells (hiPSCs) into retinal endothelial‐like cells using Wnt and Norrin signaling cues,^188^ and into brain microvascular endothelial‐like cells through Suppressor of Mothers Against Decapentaplegic (SMAD) and Wnt pathway modulation.^187^ These differentiated cells exhibit enhanced barrier function, appropriate junctional and transporter profiles, and, in some cases, the capacity to reproduce disease‐relevant changes within microfluidic systems. Further studies indicate that arterial or venous identity can be promoted by modulating VEGF, Notch, Phosphoinositide 3‐Kinase (PI3K), and Extracellular Signal‐Regulated Kinase (ERK) signaling during differentiation, followed by exposure to shear stress levels characteristic of the target vascular bed, which may help align cellular phenotype with the intended tissue.^189^, ^190^ Concurrently, there is increasing recognition that sex, ancestry, and comorbidities influence vascular phenotypes and drug responses. Several iPSC biobanking and organoid initiatives are expanding donor diversity to ensure that preclinical models more accurately reflect patient populations, rather than a limited group of young, healthy, predominantly European‐ancestry donors.^191^ While combining these advances in cell sourcing with improved materials and flow control cannot eliminate all technical barriers, they are expected to facilitate the progression of vessel‐chip models toward regulatory approval.

## 8 POSITIONING VESSEL‐CHIP PLATFORMS AS IND‐ENABLING DRUG DEVELOPMENT TOOLS

Advancing vessel‐chip technology from its current academic‐research status toward FDA‐approved preclinical tool status may require careful consideration of regulatory requirements by all stakeholders. The FDA Modernization Acts 2.0 and 3.0 have changed the rulebook by allowing non‐animal methods, such as MPS, to be used alongside or instead of animal models in IND submissions. This regulatory shift encourages vessel‐chip developers to transition from research prototypes toward tools relevant in clinical settings.^15^, ^192^ The FDA has supported this shift by establishing the ISTAND pilot program, which gives clear feedback on regulatory readiness and sets standards for qualifying biomarkers or assays in preclinical models.^193^ To make the most of this, vessel‐chip researchers now have the opportunity to include the FDA's COU criteria early in the design and validation process.^192^ Being more specific about the COU can accelerate regulatory progress. As an example, the COU for a vessel‐chip model to study “clotting disorders” could be improved by specifying that the model will be used to evaluate the risk of anticoagulant‐related hemorrhagic complications (specific clinical use) in Caucasian men over the age of 50 (specific target demographic).^194^

Getting a drug‐development tool like a vessel‐chip into the ISTAND program involves three main steps. First, a letter of intent is submitted, explaining the COU and the specific unmet need in drug development that the vessel‐chip addresses. If accepted, the next step is to work with the agency to create a qualification plan. This plan must provide evidence for both analytical and clinical validation, in accordance with the FDA's biomarker qualification framework.^195^ Analytical validation requires evidence demonstrating the model's reproducibility and reliability. Vessel‐chip assays, such as endothelial barrier permeability, platelet aggregation rate, and activation marker expression, should be supported by calibration data, appropriate controls, and standardized protocols. The results are expected to remain consistent across different chip batches, test sites, donors, and other anticipated sources of variability. Clinical validation requires evidence linking the model's biomarkers to relevant clinical outcomes and can be conducted either retrospectively or prospectively. The retrospective approach relies on using existing drugs, while the prospective approach uses drugs currently undergoing clinical trials. In the context of the previous example, a retrospective approach may use anticoagulants with well‐established bleeding risk profiles, whereas a prospective approach may examine investigational anticoagulants currently undergoing clinical trials and determine whether vessel‐chip biomarkers correlate with and accurately classify drugs into clinically observed bleeding risk categories.

The strength of the IND support package is assessed throughout the validation process and balanced against the risk of the proposed use. In high‐risk cases, such as requests to skip animal studies, the framework calls for more thorough clinical and analytical validation, which should be planned with the regulatory agency. The last step is to submit the full qualification package, including all data from the qualification plan, to the FDA. The agency then decides if the model will be accepted into the ISTAND program. For example, Emulate's Liver‐Chip S1 went through this process.^196^ This organ‐on‐chip platform was approved for predicting drug‐induced liver injury (DILI) because it had a clear COU and a plan showing that its results could identify toxic drugs in a set with known clinical outcomes.^197^, ^198^ This method enabled Emulate to integrate its platform into the biomarker qualification process, which could also serve as a good example for vascular models. While several vessel‐chip platforms are well positioned for IND readiness, establishing a narrow COU, analytical validation strategy, and high‐throughput compatibility would support their adoption as drug development tools (Table 2).

**TABLE 2:** Comparative summary and investigational new drug‐readiness of different vessel‐chip platforms.

Platform/model	Disease/application	Key design features	Material	Cell source	Key strengths	Key limitations
AIM Biotech identX BBB Model^187^, ^199^	Blood–brain‐barrier permeability	40‐assay in well plate format, microfluidic, no flow, liquid handler compatible	Cyclic olefin polymer/copolymer	iPSC‐derived ECs, pericytes, astrocytes	High throughput, multi‐cellular, standardized formulations	Broad COU definition, lacks flow control, lacks comprehensive validation.
MIMETAS OrganoReady® Blood Vessel^200^	Snake venom caused vascular damage	64‐assay in well plate format, microfluidic, gravity‐driven flow, liquid handler compatible	Polystyrene	Primary ECs (HUVEC)	Narrow COU definition, Automated readout, high throughput	High variability,^201^ lacks validation, non‐physiological substrate
Microengineered blood vessel‐on‐chip^22^	Antibody‐induced thrombosis	Microfluidic, flow control, blood perfusion, platelet aggregation, microscope slide format	PDMS	Primary ECs (HUVEC)	Narrow COU definition, clinically relevant biomarker, donor variability analysis	Low throughput, lacks analytical and clinical validation.
SCD vascular model with BOECs^105^	Thrombosis in sickle cell disease (SCD) vessel	Microfluidic, flow control, microscope slide format, blood perfusion, platelet aggregation	PDMS	Primary patient‐derived blood outgrowth EC	Clinically relevant biomarker, patient‐specific cells	Broad COU definition, low throughput, needs comprehensive validation.
Thrombo‐inflammation on chip model^202^	Thrombus resolution	Microfluidic, flow control, immune cells, blood perfusion, platelet aggregation	PDMS	Primary ECs (HUVEC)	Clinically relevant biomarker, long‐term, drug study inclusion	Broad COU definition, low throughput, needs comprehensive validation.
3D thrombosis model from CT angiography^73^	Stenosis associated thrombus formation	Microfluidic, flow control, clinically obtained vessel architecture, blood perfusion, platelet aggregation	PDMS	Primary ECs (HUVEC)	Clinically relevant biomarker, patient‐specific, physiological geometry.	Missing COU definition, low throughput, non‐standard device format, needs comprehensive validation.
Microengineered human vein‐chip model^29^	Deep vein thrombosis (DVT)	Microfluidic, flow control, blood perfusion, platelet aggregation	PDMS	Primary ECs (HUVEC)	Clinically relevant biomarker, physiological substrate and geometry	Broad COU definition, low throughput, needs comprehensive validation.
3D Atherosclerosis on a chip model^58^	Atherosclerotic plaque formation	Microfluidic, flow control, immune cells, multi‐cellular model	PDMS	Primary ECs (HUVEC), hAoSMC, THP‐1	Physiological substrate, comprehensive readouts	Missing COU definition, low throughput, low clinically relevance of the readout

Abbreviations: 3D, three‐dimensional; BOECs, blood‐derived outgrowth endothelial cells; COU, context of use; ECs, endothelial cells; iPSC, induced‐pluripotent stem cell; VSMC, vascular smooth muscle cells.

Currently, the practical integration of vessel‐chip platforms into commercial‐scale drug development pipelines is still in early stages, and there are a few limitations that need to be overcome. For example, human cell sources and reagents have inherent variability, and in some cases, organ‐specific cell sources are unavailable for cell culture or may be so expensive that practical and scalable use is not possible in a typical NIH‐funded laboratory or a small biotech company. There is also no consensus about the list of drugs that could serve as a benchmark for evaluating the predictive power of various vessel‐chip platforms, and obtaining such a consensus for COU‐specific qualification plan remains challenging.^203^ Limited standardization guidelines and resulting incompatibility of vessel‐chip platforms with legacy high‐throughput assays also pose a practical barrier for translation. However, with the recent push and encouragement by NIH and FDA to support NAMs, for example, the creation of an NIH‐wide standardized organoid modeling center (SOM) enhances access and provides protocols to control cell‐source variability. Moreover, the unique ability of microphysiological platforms to capture cell source variability is a strength of such models, not a weakness, as such variability may be present in clinical trials but is not captured by animal models. Emerging consensus among organ‐chip stakeholders, including the FDA, National Institute for Standards and Technology (NIST), and other multinational organizations, is paving the way for clear standardization guidelines to accelerate translation.^204^, ^205^, ^206^

## 9 CONCLUSION

With recent advances in 3D bioprinting, microfabrication, and tissue engineering, it is now possible to recreate complex vascular architectures, such as stenosis,^55^, ^207^ bifurcations,^74^ and venous valves,^29^ that more accurately mimic human physiology. These anatomically relevant vessel‐chip models allow precise investigation of CVDs, including atherosclerosis and DVT, by enabling replication of pathological hemodynamic conditions such as recirculating and oscillatory flows found in deep veins and carotid arteries. The integration of sophisticated microfluidic systems further enhances the ability to study endothelial responses under dynamic shear stress environments.^119^, ^181^

Parallel to these engineering advancements, the advent of iPSCs, following the discovery of Yamanaka factors, has transformed the field of disease modeling. Directed differentiation of iPSCs into ECs, alongside alternative sources like EPCs and BOECs, has enabled the development of disease, population, and patient‐specific vessel‐chip platforms.^85^, ^106^ These platforms provide renewable, autologous, and phenotypically relevant models to investigate vascular dysfunction in both common and rare diseases, including T1D, SCD, and HGPS.^116^, ^117^

Vessel‐chip technology, when integrated with patient‐derived vascular cells, offers a transformative and human‐relevant platform for studying endothelial dysfunction, vascular inflammation, and disease progression. The incorporation of multi‐omics approaches (e.g., transcriptomics and proteomics) and AI‐driven analytics significantly enhances the predictive power of these models. Real‐time imaging and high‐content data sets enable mechanistic insights, patient stratification, and personalized drug testing—ultimately accelerating drug discovery and improving clinical trial outcomes. As these systems evolve over the next few years and are validated against orthogonal experimental models and clinical data sets, they are expected to undergo standardization and a more widespread acceptance within the preclinical research pipelines of the pharmaceutical industry. Ultimately, while limitations remain that may be addressed through interdisciplinary collaborations, sustainable funding and resource allocation, and involvement of the pharmaceutical industry, the prospective value of vascular organ‐chips is compelling as they offer the promise of enabling precision interventions and treatment strategies, ultimately resulting in FDA IND approvals relatively faster and lower financial burden over the next decade.

## AUTHOR CONTRIBUTIONS

AK, RM, and TM designed the research strategy along with AJ, and conducted the primary literature search, screening, and selection of relevant articles. AK, RM, and TM extracted and organized key information from the included studies, analyzed and synthesized the evidence, and drafted the initial version of the manuscript. JPC, AM, J‐iA, N‐TL, GW, and YX provided critical intellectual input on the authorship, interpretation and integration of the literature, AJ, conceived the overall concept and structure of the review, and supervised the direction and progress of the project. All authors contributed to refining the arguments and presentation, and all authors reviewed and approved the final version of the manuscript.

## FUNDING INFORMATION

This material is based upon work supported by the US Army Medical Research (USAMRAA) Contract No. HT94252410432; NASA, BARDA, NIH, and United States Food and Drug Administration (USFDA), under Contract No. 80ARC023CA002 to John P. Cooke and Abhishek Jain; NHLBI of NIH under Award Number R01HL157790; NSF CAREER Award number 1944322; and TAMU Office of Innovation Translational Investment Funds to Abhishek Jain; and NIH grants RO1 HL148338; R01 HL157790 to John P. Cooke. S1‐META.

## CONFLICT OF INTEREST STATEMENT

There are no conflicts of interest.

## Data Availability

There is no new data created or analyzed in this article.
